# Investigating the Mechanism of Low-Salinity Environmental Adaptation in *Sepia esculenta* Larvae through Transcriptome Profiling

**DOI:** 10.3390/ani13193139

**Published:** 2023-10-08

**Authors:** Yongjie Wang, Xiumei Liu, Weijun Wang, Guohua Sun, Xiaohui Xu, Yanwei Feng, Zan Li, Jianmin Yang

**Affiliations:** 1 School of Agriculture, Ludong University, Yantai 264025, China; 2 College of Life Sciences, Yantai University, Yantai 264005, China

**Keywords:** low salinity, *Sepia esculenta*, larvae, protein–protein interaction network, RNA-Seq

## Abstract

**Simple Summary:**

The cuttlefish, *Sepia esculenta*, is both a valuable economic resource and a creature of scientific interest. Our study investigated how changes in ocean salinity, a factor subject to environmental shifts, affect young, more responsive cuttlefish. We examined the gene expression of cuttlefish when exposed to lower levels of salinity. Using transcriptomic and enrichment analysis, we identified over a thousand genes that were differentially expressed under these circumstances. The images created from the results of these analyses can be used to visualize the content of the analyses. Interestingly, we discovered that low salinity could trigger harmful cell proliferation in juvenile cuttlefish, thus disrupting their normal functions. This information is valuable as it can inform potential measures to reduce the mortality rate of hatchling cuttlefish in artificially controlled conditions.

**Abstract:**

*Sepia esculenta* is an economically important mollusk distributed in the coastal waters of China. Juveniles are more susceptible to stimulation by the external environment than mature individuals. The ocean salinity fluctuates due to environmental changes. However, there is a lack of research on the salinity adaptations of *S. esculenta*. Therefore, in this study, we investigated the differential expression of genes in *S. esculenta* larvae after stimulation by low salinity. RNA samples were sequenced and 1039 differentially expressed genes (DEGs) were identified. Then, enrichment analysis was performed using the Gene Ontology (GO) and Kyoto Encyclopedia of Genes and Genomes (KEGG) databases. Finally, a protein–protein interaction network (PPI) was constructed, and the functions of key genes in *S. esculenta* larvae after low-salinity stimulation were explored. We suggest that low salinity leads to an excess proliferation of cells in *S. esculenta* larvae that, in turn, affects normal physiological activities. The results of this study can aid in the artificial incubation of *S. esculenta* and reduce the mortality of larvae.

## 1. Introduction

For aquaculture, a stable and suitable marine environment can facilitate the development of the aquaculture industry [1,2]. Global warming has led to accelerated glacier melting in recent years [3,4]. More melting of glaciers will dilute the salt in the ocean and reduce its salinity. The decrease in ocean salinity will have a great impact on the life activities of marine mollusks [5]. In a previous study, Knowles et al. explored hemolymph chemistry and histopathological changes in Pacific oysters (*Crassostrea gigas*) under low salt stress [6]. They found significant vacuolization in the cytoplasm of the summer Pacific oyster when stimulated by low salt concentrations, leaching of blood cells from the lining of the digestive tract, and significant tissue lesions. Ren et al. sequenced and analyzed *Sepia pharaonis* larvae and found that low salinity had significant negative impacts on life processes such as fatty acid metabolism, ion transport, and osmotic pressure regulation [7].

High-throughput transcriptome sequencing is a technology that has developed relatively rapidly. The analysis of organism activities has become more accurate and efficient using this technology [8,9,10]. Previous studies have used this technique to examine biological processes in mollusks. For example, using transcriptome analysis, Boamah et al. found that heat shock protein 90 (HSP90) was significantly up-regulated and tumor necrosis factor (TNF) was significantly down-regulated in *Haliotis discus hannai* in response to low salt stimulation and that immune responses were activated [11]. Transcriptome profiling of *Amphioctopus fangsiao* at different time points by Bao et al. revealed the presence of an immune response mechanism for the protection of eggs [12]. Therefore, transcriptome sequencing technology was utilized in the present study to probe the biological processes that function in a mollusk following low-salinity stimulation.

*Sepia esculenta* is a species of cuttlefish with high nutritional value and commercial significance. The species is distributed in the waters of Eastern China, Korea, and Japan [13]. *S. esculenta* can be affected by fluctuations in salinity during its growth and development. The larvae have a lower ability to withstand environmental stresses compared with the adults [14]. It also exhibits higher mortality rates when subjected to extreme environmental stresses, which is extremely detrimental to the aquaculture industry. Therefore, larvae after the completion of incubation were used as experimental samples in this study to better understand the effects of low salinity on mollusks.

In this study, sequencing was used to investigate the biological processes of *S. esculenta* stimulated by low salinity. *S. esculenta* exposed to low salinity were sampled and sequenced at 0, 4, and 24 h, and the data were mapped to the reference genome. Differentially expressed genes (DEGs) were screened and analyzed using DESeq2. The DEGs were then used for GO and KEGG enrichment analyses, and protein–protein interaction networks (PPI) were constructed. The results of PPI and KEGG enrichment analyses were used in a combined analysis to explore the expression of DEGs in *S. esculenta* larvae after low-salinity stimulation. Finally, quantitative RT-PCR was used to validate the quality of the sequencing results. The results of this study reveal the effects of low-salinity stimulation on the growth and development process of *S. esculenta* larvae, which can be helpful for further investigating the resistance mechanism of larvae in extremely unfavorable environments. Meanwhile, it can also support researchers who need to conduct larval breeding in response to changes in the marine environment.

## 2. Materials and Methods

### 2.1. Experimental Samples and Procedures

Adult cuttlefish from the sea in the Qingdao area were used in this study. The adults obtained were placed in a saltwater tank for short-term culture to ensure normal growth in the saltwater tank environment. The water temperature of the temporary breeding pool was 21.5 ± 1 °C. Attachment nets were placed in the pools, and the eggs were collected daily and placed in pots with small holes. The eggs were oval in shape, 6.5 ± 0.5 mm in diameter, and translucent. Fertilized eggs were incubated at 21.5 ± 1 °C and the membranes were successively removed and hatched out after 26 d. The basin with the eggs was placed in the pool to ensure the flow of seawater and provide sufficient oxygen. We ensured that the chemical properties of the water such as pH, temperature, and dissolved oxygen were the same as in the mother saltwater tank (Table S1).

The experiment was conducted using two buckets, each with a capacity of 120 L and containing 100 L of water. Larval samples were immediately transferred to prepared experimental buckets half an hour after the completion of incubation. The salinity before transfer was 30 ppt. In previous studies, mollusks in a 20 ppt salinity environment produced significant immune defenses [15]. Therefore, the salinity of the treatment group was set to 20 ppt, and the salinity of the control group remained unchanged at 30 ppt. In the experiment, 100 *S. esculenta* larvae were placed in each bucket and sampled at 4 and 24 h, with the 0 h sample being obtained separately. Nine larvae were randomly selected at each time point and every third larva was mixed to form three replicates. All samples obtained were placed in tubes and frozen in liquid nitrogen for storage.

### 2.2. Detection of Sample RNA

The RNA extraction and RNA-Seq were performed by Novogene. RNA was extracted from whole individual hatchlings using the TRIzol method and subjected to stringent quality control utilizing an Agilent 2100 bioanalyzer [16]. A NEBNext^®^ Ultra™ Directional RNA Library Prep Kit was used for library construction. The nine larvae were randomly selected from the 4 h and 24 h low-salinity and control groups and three larvae were randomly pooled to form three replicates. An Illumina NovaSeq 6000 was used for sequencing.

### 2.3. Analysis of DEGs

The quality of the sequencing results was assessed using FastQC software (version 0.12.0) [17]. We filtered the raw reads to remove reads with adapter, to remove reads with a proportion of N greater than 10%, and to remove low-quality reads (those with a quality value Qphred ≤ 20 where the number of bases accounted for more than 50% of the whole reads). We calculated the average score for Q20 and Q30. The quality control clean reads were mapped to the reference genome by HISAT2 software (version 2.2.1) [18]. The reference genome of *S. esculenta* is currently presented as unpublished. DEGs were screened out using the DESeq2 package for R as a negative binomial distribution model. First of all, data were imported for building the ddsmodel, and then, the DESeq function was used to estimate the dispersion of the samples. Afterwards, the difference in gene expressions was analyzed by this package. DEGs with |Log2 Fold Change| ≥ 1 and *p*-value ≤ 0.01 were screened [19].

This study utilizes volcano plots, heat maps, and Venn diagrams to demonstrate the expression of DEGs. The distribution of gene expression is demonstrated by a volcano plot. The significance of the difference between the two groups of samples was shown by assigning different colors to the points with significant differences using the multiplicity of differences between the groups, with the logarithm of the base of 10 as the horizontal coordinate and the significance of the differences with the *p*-*value* of the test of significance taking the logarithm of the base of 10 as the vertical coordinate. The expression patterns of DEGs in different treatment groups are demonstrated by clustered heat maps. The FPKM data of DEGs were normalized using Z-score and selected for horizontal clustering. The FPKM values are the average expression of three biological replicates for each case. The clustering of the samples was achieved by calculating the Euclidean distances between the samples using pheatmap package of R [20]. The intersection of the number of genes expressed at different time points is illustrated by a Venn plot.

The DAVID v6.8 online site was used for functional enrichment analyses using the GO and KEGG databases [21]. The default parameters of the site were used for functional enrichment analysis of genes. The gene codes we uploaded were used in the background gene set for DAVID analysis, combined with previous studies, to identify signaling pathways that were significantly enriched to assume key responsibilities after low-salinity stress. The enrichment of DEGs into the GO term and KEGG signaling pathways enabled clarification of the mode of action of genes under low salt stress.

The STRING v11.5 online site was used for the construction of a PPI network [22]. The gene sequences of all DEGs were uploaded to STRING for homologous sequence comparison, and the highest scoring sequences were used for STRING analysis. The minimum interaction score was set to 0.150 and the other parameters were left at default. The functions of key DEGs in *S. esculenta* after low salt stress were analyzed according to the number of PPIs involved and the number of KEGG signaling pathways.

### 2.4. qRT-PCR Validation

The 16 genes identified as playing key roles following low-salinity stress in larvae were used to validate the accuracy of the sequencing results. The relative quantities of the low-salinity group (SAL20_4h) and the control group (C_4h) were compared to derive the value of fold change for 4 h. The same comparison method yielded the value of fold change for 24 h. These similar expression trends of the gene in RNA-Seq and qRT-PCR prove that the sequencing results are accurate. First, Primer Premier 5.0 [23] was used to design the gene-specific primers of key genes. To ensure the accuracy of the experiment, we compared the expression levels of three reference genes including *β-actin*, *18S*, and *GAPDH* in *S. esculenta* larvae before experiment, and *β-actin* was selected because of its stable expression. Then, quantitative RT-PCR was performed in a 20 µL solution containing 10 ng of template cDNA and SYBR Premix Ex Taq II (TaKaRa) by using a LightCycler 480 at 95 °C for 5 min pre-incubation, followed by 45 cycles of 95 °C for 15 s and 60 °C for 45 s. Finally, the melting curve was analyzed to detect single amplification. Fluorescent signal accumulation was recorded at the 60 °C 45 s phase during each cycle by using LightCycler 480. The 2^−ΔΔCt^ comparative Ct method was used to calculate the relative quantities of the target genes expressed as fold variation over *β-actin* (Table S2) [24].

## 3. Results

### 3.1. Sequencing Results

In this study, transcriptome technology was used to analyze and probe the low-salinity response in *S. esculenta*. An analysis of the results obtained from sequencing showed that the Q20 scores were all above 95%, and the average score for Q30 was 91.94% (Table S2). This indicated that the sequencing results were satisfactory.

### 3.2. Analysis of DEGs

**Figure 1:**
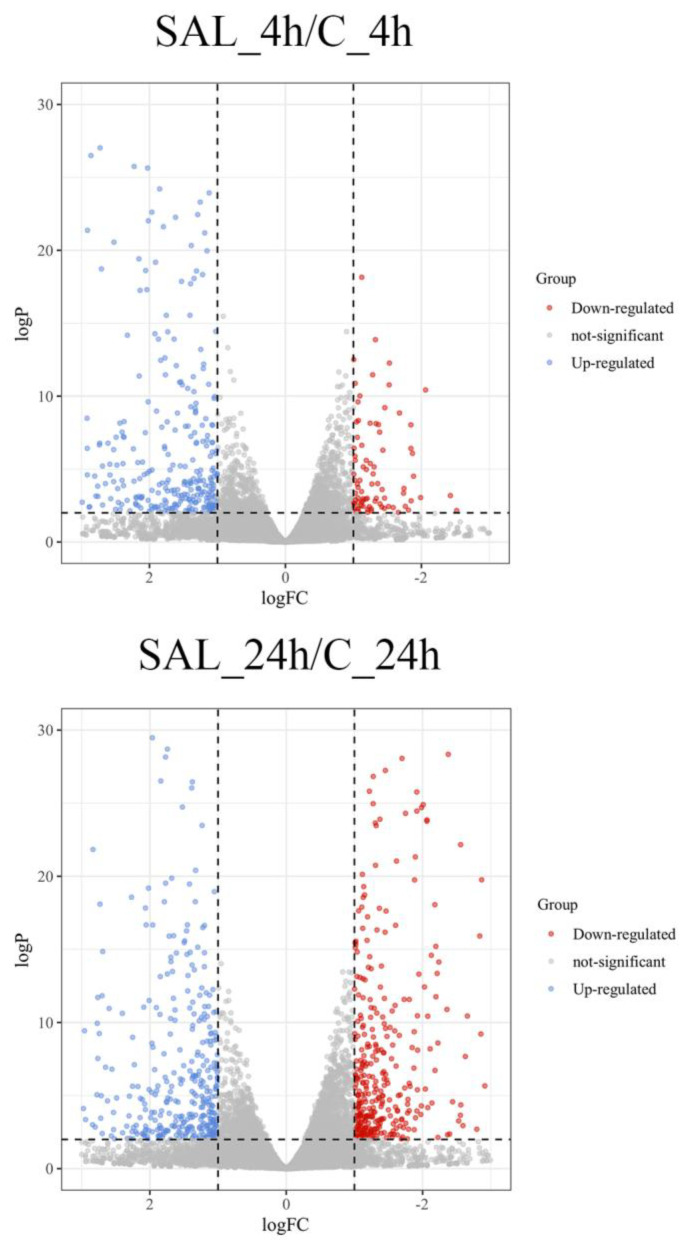
Volcano plot of the distribution of the expression levels of genes. Each dot represents a gene. Red dots indicate differentially down-regulated genes; blue dots indicate genes that were differentially up-regulated; and gray dots indicate genes that were not significantly different.

In this study, our DEG results are presented using volcano plots, clustered heat maps, and Venn plots. The volcano map results showed that 433 DEGs were identified in the 4 h exposure samples. Among all differentially expressed genes, 342 were up-regulated, and 91 genes were down-regulated. Among the 24 h exposure time samples, 764 genes were significantly differentially expressed; 422 DEGs were up-regulated, and 342 DEGs were down-regulated (Figure 1).

**Figure 2:**
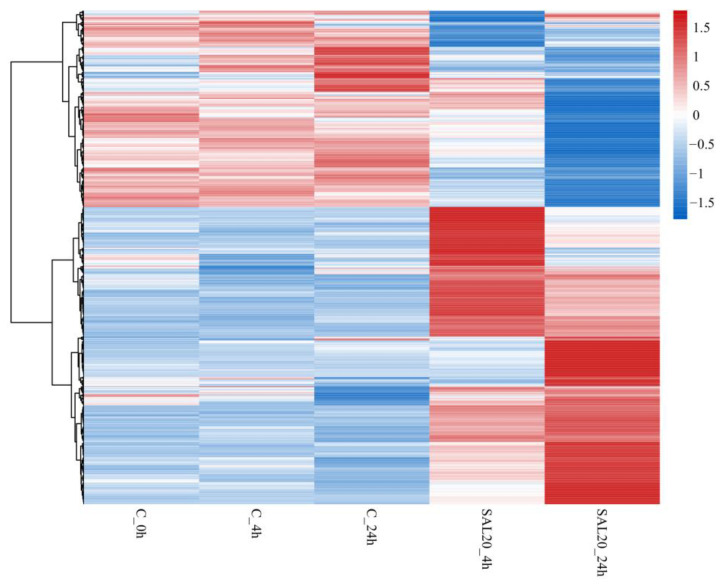
Hierarchical clustering heat map of DEGs. The color ranges from red to blue, indicating differential expression levels from high to low. Each column represents a different treatment group. Each row represents a gene. The clustering tree shows all significantly differentially expressed genes.

The clustering heat map reflected the overall expression of the sample. The results showed that the expression profiles of the three control groups were essentially the same (C_0h, C_4h, and C_24h). The expression patterns of the two experimental groups (SAL20_4h and SAL20_24h) showed significant differences compared with the control group with the same exposure time (Figure 2).

**Figure 3:**
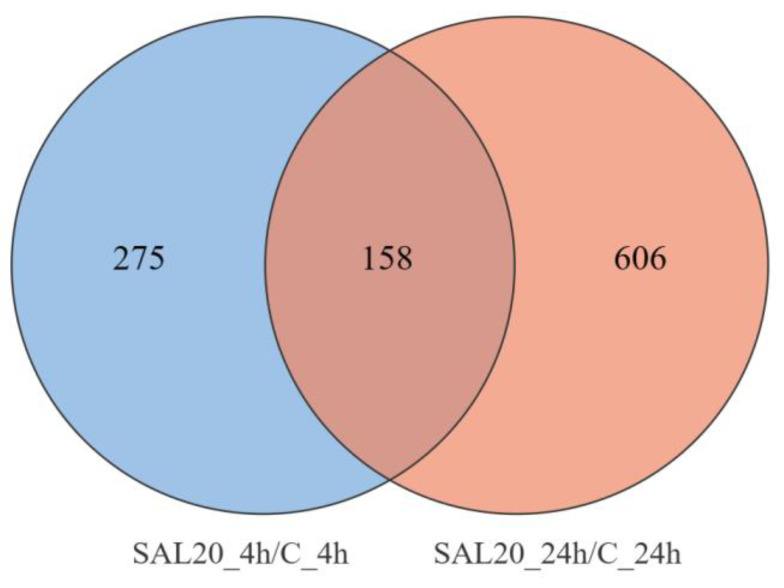
Venn diagram of DEGs. Blue and orange colors indicate the numbers of differentially expressed genes at 4 and 24 h, respectively. The number of co-expressed genes is indicated by the dark-brown area.

The Venn plot showed that 764 genes were significantly differentially expressed at 24 h of exposure time, and 433 genes were significantly differentially expressed at 4 h of exposure time. There were 158 DEGs that showed significant differential expression at both 4 and 24 h (Figure 3).

### 3.3. Analysis Using GO and KEGG

**Figure 4:**
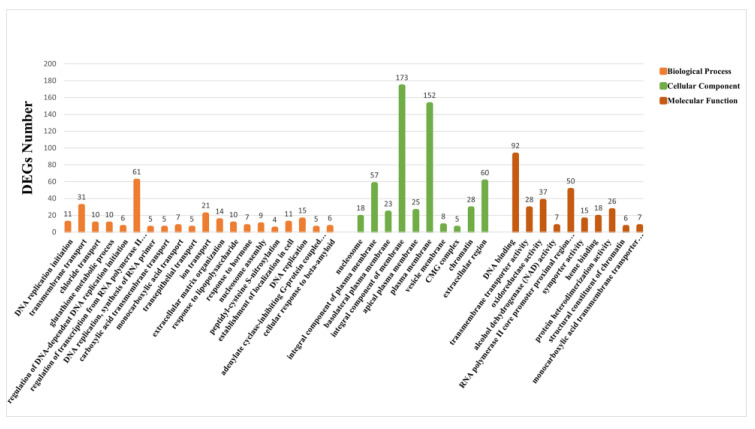
GO analysis of DEGs. The different colors represent the different categories from the GO database.

DEGs were enriched in three categories in the GO: biological processes, cellular components, and molecular functions. The top 20 terms for biological processes and cellular components and the top 10 terms for molecular functions were selected (*p* ≤ 0.01) (Figure 4). In the first 20 terms of biological processes, processes such as “transmembrane transport” and “ion transport” were related to the transfer and transport of substances.

**Figure 5:**
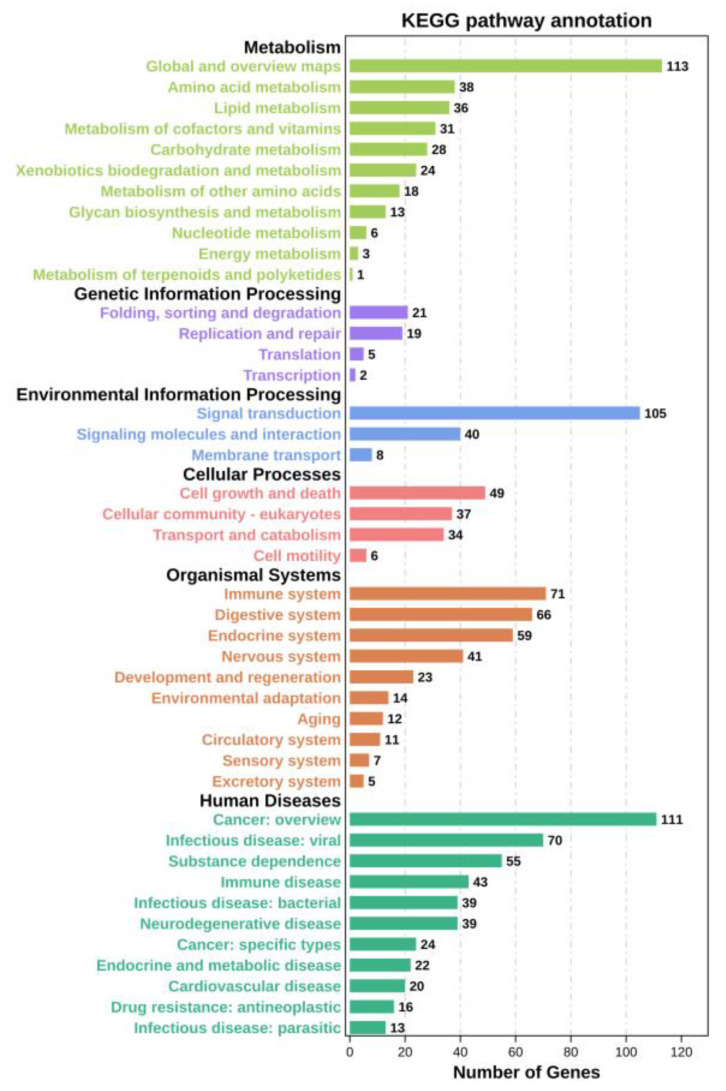
Significant enrichment of related signaling pathways in Level-2.

The analysis of the KEGG results revealed that most of the signaling pathways were related to intercellular substance transport (Figure 5). Sixteen of the signaling pathways that were significantly enriched after low-salinity exposure were screened (Table 1).

### 3.4. Analysis of PPI Network Results

**Figure 6:**
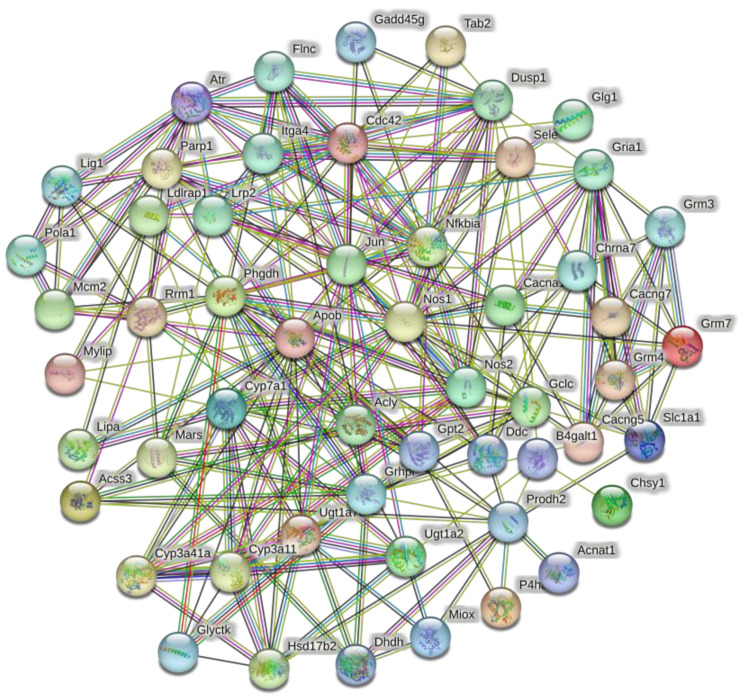
Low-salt-stress-related PPI network. Each node represents a gene, and the number of edges between genes represents the number of interacting relationships.

Sixty-one DEGs from 19 signaling pathways that were enriched after low salt exposure were used to construct the PPI network (Figure 6). The relevant parameters for the PPI network were calculated (Table 2). The data related to PPI parameters indicated robust interrelationships between DEGs.

The number of genes involved in interactions with PPIs was used as the primary reference factor, and the number enriched to the KEGG signaling pathway was used as a secondary reference factor to identify key genes that function after low-salinity stress. Sixteen key DEGs were finally identified. Table 3 lists the 16 identified key DEGs and the corresponding numbers from the PPI and KEGG analyses.

### 3.5. Analysis by qRT-PCR

**Figure 7:**
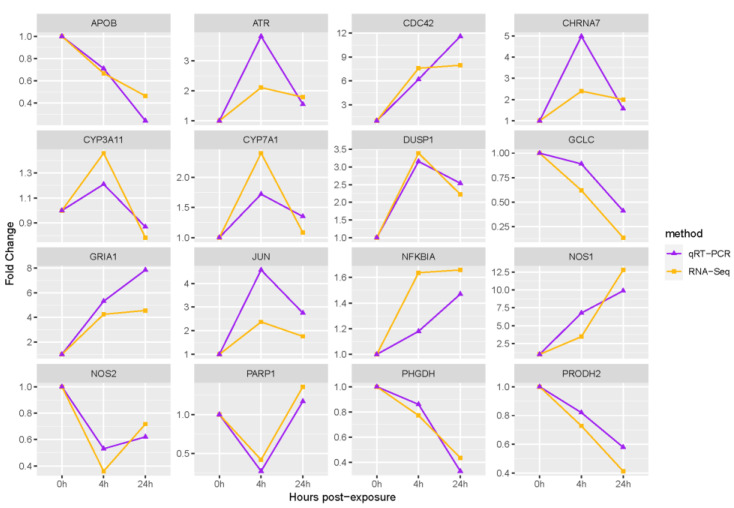
Quantitative validation of key genes.

The expression of the identified key genes was verified by qRT-PCR. Analysis of the qRT-PCR results indicated that the expression trends of the genes sequenced at different time points were consistent with those of the quantitative validation, suggesting that the sequencing results were reliable (Figure 7).

## 4. Discussion

### 4.1. Enrichment Analysis of DEGs

Too-low salinity can be fatal to mollusks [5]. In the present study, 1039 significantly differentially expressed DEGs were screened in *S. esculenta* stimulated by low-salinity seawater. The results illustrated in the volcano and Venn diagrams demonstrate a significant increase in differentially expressed genes with increasing exposure time, suggesting that the low salinity had severe effects on *S. esculenta* larvae. The clustering heat map results indicated that the 4 h exposure group (SAL20_4h) and the 24 h exposure group (SAL20_24h) showed significant differential expression patterns compared with the control group (C_4h, C_24h) with the same exposure time.

In this study, 1039 DEGs showing significant differential expression were used for GO and KEGG functional enrichment analyses. The GO enrichment analysis revealed that most of the 20 terms identified in the biological processes are related to the transportation of substances. The significant enrichment of “transmembrane transport” in the classification of biological processes can significantly enhance the exchange of substances between cells and tissues, effectively reducing the stimulation of organisms in response to environmental changes and maintaining cellular homeostasis [25,26,27,28]. “Ion transport” is a very common biological process in the growth and metabolism of mollusks. Through this process, mollusks can regulate the osmotic pressure of their body, and abnormal osmotic pressure can lead to the death of the organism [29,30,31]. This process was significantly enriched in the GO analysis, suggesting that low salinity can lead to abnormal osmotic pressure in *S. esculenta* larvae [15]. Of the 16 signaling pathways significantly enriched in the KEGG functional analysis, many were significantly associated with metabolic and immune responses. The MAPK signaling pathway and the Toll-like receptor signaling pathway are important in the immune response of mollusks [32,33,34,35]. Cholesterol metabolism can significantly affect the growth and development of mollusks [36,37]. The GO and KEGG enrichment analyses revealed that low-salinity seawater has the potential to affect the growth and development of *S. esculenta* larvae. These results can be applied to the artificial breeding of *S. esculenta* in response to environmental changes such as rainfall-induced reductions in salinity.

### 4.2. Combined Analysis of KEGG and PPI

The growth, development, reproduction, and other life activities of living organisms are supported by proteins [38,39,40]. In this study, 61 genes significantly enriched in 16 KEGG signaling pathways were used to construct a PPI network for *S. esculenta* larvae experiencing low-salinity stress. Analysis of the PPI results demonstrated a highly significant interaction between differentially expressed proteins. For example, the number of protein interactions between JUN (Jun proto-oncogene, AP-1 transcription factor subunit) and APOB (apolipoprotein B) was greater than 20. By analyzing the PPI results, we conclude that the abovementioned proteins play important roles in the response of *S. esculenta* larvae to low salinity. The specific functions assumed by the differentially expressed proteins during low-salinity stimulation need to be further analyzed and explored. By synthesizing the results of PPI and KEGG analyses, 16 genes that play a significant role were identified in *S. esculenta* stimulated by low salinity.

#### 4.2.1. Analysis of Inflammatory and Immune Responses

Unlike vertebrates, mollusks do not have acquired immunity and rely only on innate immunity to protect themselves from external stimuli [41,42]. Therefore, the activated immune response in invertebrates is particularly important when stimulated by environmental factors. The immune system is made up of cells and proteins responsible for immune defense in living organisms, and their coordinated responses to non-self-substances are called immune responses [43,44]. When the organism is stimulated by the external environment, it will produce an immune response, and at the same time, an inflammatory response will also occur [45,46,47]. An inflammatory response is a typical phenomenon in which an organism reacts to a biological, chemical, or physical stimulus [48]. The occurrence of inflammation in organisms means that damaged tissues and cells begin to be cleared and homeostasis is restored in the body [49,50]. Previous studies have pointed out that external stimuli can cause the NF-kappa B signaling pathway, NOD-like receptor signaling pathway, and other classical signaling pathways in *Haliotis discus hannai* to be significantly activated [51]. In our study, DUSP1 and NFKBIA were significantly enriched for involvement in the immune response. DUSP1 is an enzyme that removes phosphate groups from phosphatases and is able to regulate a variety of immune-related signaling pathways [52,53]. NFKBIA belongs to the NF-kappa B family and has a central role in coordinating various immune and inflammatory responses [54]. In our results, the expression levels of DUSP1 and NFKBIA were significantly up-regulated after low-salinity stimulation. Based on these results, we can speculate that low-salinity stimulation significantly activates the immune defense of larvae. However, the immune response mechanism of *S. esculenta* has not been fully investigated and needs to be further explored.

#### 4.2.2. Abnormalities in Metabolism

Metabolic processes are important biological processes in organisms that provide energy for cellular functioning. Abnormalities in metabolism may lead to irreversible damage to cells and tissues [55,56,57]. Lipid metabolites can also be regulators of protein function and cellular signal transduction [58]. In this study, five key DEGs were identified as having a strong correlation with metabolism, namely CYP7A1, CYP3A11, APOB, PHGDH, and PRODH2. Abnormal expression of CYP7A1 leads to a disruption of cholesterol metabolism that, in turn, leads to cellular damage [59]. Overexpression of PHGDH can regulate the synthesis of nicotinamide to maintain lipid homeostasis [60]. This gene was significantly down-regulated in the present study, suggesting that abnormal lipid metabolism occurred in our young larvae after stimulation. In this study, the above genes were significantly differentially expressed, suggesting that low salinity led to abnormal metabolism in *S. esculenta* larvae.

#### 4.2.3. Signal Transduction was Enhanced by Low Salinity

Almost all biological processes require the participation of signal transduction [61,62]. In the present study, four DEGs, CHRNA7, NOS1, NOS2 and GRIA1, were identified as significantly enhanced signal transduction-related genes. The α7 neuronal nicotinic acetylcholine receptor gene (CHRNA7) and glutamate ionotropic receptor AMPA type subunit 1 (GRIA1) have complex regulatory mechanisms and are commonly expressed in organisms [61,62]. NOS1 and NOS2 belong to the nitric oxide synthase family and play a role in several biological processes, including neurotransmission and signal transduction [63]. NOS2 enhances the immune response of an organism [64]. The expression level of this gene was significantly up-regulated, which suggested that immune defense was activated in juveniles after being stimulated by low salinity. Analysis of these results demonstrates that biological processes such as immune defenses may be significantly activated in larvae when stimulated by low salinity to defend themselves against damage caused by external stimuli.

### 4.3. Analysis of Two Hub Genes

In this study, two genes, APOB and JUN, were identified as pivotal genes that play critical roles in *S. esculenta* when stimulated by low salinity. Apolipoprotein B is the main structural protein of low-density lipoprotein (LDL-C), whose main function is to bind and transport lipids to various parts of the organism for metabolism and other biological processes [65]. JUN is a highly conserved proto-oncogene that is under-expressed or not expressed when the organism is functioning normally. When activated by foreign substances or environmental stimuli, it becomes highly expressed, leading to abnormal cell proliferation [66,67]. In our results, these two genes have the most PPI interactions. We hypothesize that the effects of low salinity on larvae are dramatic, activating immune defenses and generating inflammation, leading to abnormalities in metabolic homeostasis.

### 4.4. Exploring the Functions of Other Key DEGs

There were also genes bearing important responsibilities that were identified as key genes after low-salinity stress. GCLC promotes the synthesis of glutathione that in turn enhances the antioxidant defense of cells and regulates cell proliferation [68]. PARP1 is a marker of immune status that responds to the inflammation status in the organism [69]. The fact that these genes showed significant differential expression also suggests that *S. esculenta* larvae have enhanced antioxidant defenses and develop an inflammatory response when stimulated by low salinity, demonstrating that the effect of low salinity on the larvae is dramatic.

## 5. Conclusions

In this study, we explored the differential expression of genes in *S. esculenta* larvae after low-salinity stimulation using transcriptome sequencing. APOB is closely related to significant differences in the level of gene expression, suggesting that in larvae under low salinity, material transfer is significantly enhanced. Combined with the analysis of immune signaling pathways, which may be immune defense of the larvae, it was found that significantly enhanced material transport strengthened the immune response. The differential expression of genes involved in metabolism and signal transduction suggested that metabolism was accelerated in larvae when stimulated by low salinity, which may also play a role in enhancing immune defense. Taking all the results together, we hypothesized that low-salinity stimulation would lead to significant immune defense in juveniles, and at the same time, metabolically related signaling pathways were enhanced to provide assistance for the immune response. Therefore, it is necessary to pay attention to the damage caused by extreme environmental stimuli to larvae during the artificial breeding period.

## Figures and Tables

**Table 1:** Sixteen KEGG signaling pathways.

Pathway	Number of DEGs
Apoptosis	4
Arginine and proline metabolism	3
Arginine biosynthesis	2
Ascorbate and aldarate metabolism	3
Cell adhesion molecules	3
Cell cycle	5
Cholesterol metabolism	6
Cholinergic synapse	2
DNA replication	6
Focal adhesion	2
Glutamatergic synapse	6
Glycine, serine, and threonine metabolism	3
MAPK signaling pathway	8
Metabolic pathways	27
NOD-like receptor signaling pathway	3
Toll-like receptor signaling pathway	2

**Table 2:** Statistics of low-salt-stress-response-related PPI network parameters.

Stats	
Number of nodes	54
Number of edges	245
Average nodes	9.07
Clustering coefficient	0.558
Number of expected edges	164
*p*-value	1.83 × 10^−9^

**Table 3:** Key DEGs and the results from the PPI and KEGG analyses.

Gene	Gene Name	Number of PPI	Number of KEGG
*APOB*	apolipoprotein B	23	3
*ATR*	ATR serine/threonine kinase	11	1
*CDC42*	cell division cycle 42	17	4
*CHRNA7*	cholinergic receptor nicotinic alpha 7 subunit	12	1
*CYP3A11*	cytochrome P450, family 3, subfamily a, polypeptide 11	11	5
*CYP7A1*	cytochrome P450 family 7 subfamily A member 1	15	2
*DUSP1*	dual specificity phosphatase 1	15	2
*GCLC*	glutamate-cysteine ligase catalytic subunit	13	2
*GRIA1*	glutamate ionotropic receptor AMPA type subunit 1	14	2
*JUN*	Jun proto-oncogene, AP-1 transcription factor subunit	26	6
*NFKBIA*	NFKB inhibitor alpha	17	5
*NOS1*	nitric oxide synthase 1	18	4
*NOS2*	nitric oxide synthase 2	14	4
*PARP1*	poly(ADP-ribose) polymerase 1	12	1
*PHGDH*	phosphoglycerate dehydrogenase	18	2
*PRODH2*	proline dehydrogenase 2	12	1

## Data Availability

We have uploaded and published our raw data in the Sequence Read Archive database on NCBI. The SRA accession numbers are SRR23936169, SRR23936170, SRR23936171, SRR23936172, SRR23936173, SRR23936174, SRR23936175, SRR23936181, SRR23936182, SRR25097168, SRR25097169, SRR25097170, SRR25097171, SRR25097172, and SRR25097173. The specific website for querying these data is: https://www.ncbi.nlm.nih.gov/Traces/study/?acc=PRJNA947123&o=library_name_s%3Aa%3Bacc_s%3Aa (accessed on 25 September 2023).

## Supplementary Materials

The following supporting information can be downloaded at: https://www.mdpi.com/article/10.3390/ani13193139/s1, Table S1: Experimental seawater parameter; Table S2: Primers used for qRT-PCR; Table S3: RNA-Seq sequencing results and quality analysis.
