# Supplementary material for: Investigating the Mechanism of Low-Salinity Environmental Adaptation in Sepia esculenta Larvae through Transcriptome Profiling
Source: Animals (Basel). 2023 Oct 8;13(19):3139. doi: 10.3390/ani13193139 (PMC10571815; doi:10.3390/ani13193139)
Supplement: Supplementary file 1 [file animals-13-03139-s001.zip › Table S1.pdf]

Table S1. Experimental seawater parameter.

seawater parameter	concrete data
ocean temperature	21.5 ± 1℃
Control salinity	30 ± 0.3 ppt
Treatment group salinity	20 ± 0.3 ppt
pH	8.2
Dissolved Oxygen	5.5mg/L
