# Supplementary material for: Investigating the Mechanism of Low-Salinity Environmental Adaptation in Sepia esculenta Larvae through Transcriptome Profiling
Source: Animals (Basel). 2023 Oct 8;13(19):3139. doi: 10.3390/ani13193139 (PMC10571815; doi:10.3390/ani13193139)
Supplement: Supplementary file 1 [file animals-13-03139-s001.zip › Table S2.pdf]

Table S2. Primers used for qRT-PCR.

Gene name	Forward primer (5′-3′)	TM (°C)	Reverse primer (5′-3′)	TM (°C)	Amplicon length (bp)
APOB	TACTGACTGCTCTGCTACTT	60	GCCACCGCTTCGTATTT	60	121
ATR	GGAAGGTCAAGGCTCTAAAC	60	CATGTACACACAGAGGAAGAC	60	105
CDC42	CTCAGTTATACGACCAGAGGA	60	GCCGTGTCAACGAGATTTA	60	116
CHRNA7	CCATCATCACCACCATCATC	60	TCTCCTCCTCCGACTATCT	60	108
CYP3A11	ACAGACCTCCTCTACCATATC	60	TTTCTCGCGCTGTAAGATG	60	118
CYP7A1	CTCACTTTCGGTACCCTTTG	60	CGTGACCGTGGTAGTTAATG	60	142
DUSP1	GTCTTTCTCGGTGACCATTG	60	GGAAGAGAGATCGGCATACT	61	165
GCLC	CCCAGGGAAACCATTTAGTAG	60	AGAGTCACAAGTGCTTCATC	60	117
GRIA1	GATTGTGGAGTTCGTCTGT	59	TCATTTCCAGTCGGTGTATC	59	105
JUN	CCGAGTTGGAAAGGATGATT	60	CACAAATCCTCGGGCATAG	60	122
NFKBIA	CTGCCCTCCAGAAACATTAC	60	CCCATGTTCAGCAGCATAA	60	116
NOS1	CCCACTTGAATGTTGCTCTT	61	GCAGTCAGCACAGAGTAGA	61	103
NOS2	GACAGAAGTGGTGGACATTC	60	GCGACAGTACCTGATCTTTG	60	140
PARP1	AGGCAGTGGTGGAATAGA	60	CACGACATGTACTCCGATTG	60	113
PHGDH	TGGCTCTAAGCAGGAACA	60	CGTCCCAAACCAAGAATACC	61	121
PRODH2	CAGATGCACAGGGAGTAAAG	60	CTTGTGTCTTCGTAGGTGTC	60	124
