# Supplementary material for: Investigating the Mechanism of Low-Salinity Environmental Adaptation in Sepia esculenta Larvae through Transcriptome Profiling
Source: Animals (Basel). 2023 Oct 8;13(19):3139. doi: 10.3390/ani13193139 (PMC10571815; doi:10.3390/ani13193139)
Supplement: Supplementary file 1 [file animals-13-03139-s001.zip › Table S3.pdf]

Table S3. RNA-Seq sequencing results and quality analysis.

sample	Raw reads	Raw bases	Clean reads	Clean bases	Q20 (%)	Q30 (%)	Total mapping
C_0h_1	46113806	6.92G	44982104	6.75G	97.07	92.32	39456868
C_0h_2	50545156	7.58G	49387700	7.41G	97.44	93.02	44052010
C_0h_3	47515546	7.13G	46359916	6.95G	97.27	92.68	41025164
C_4h_1	46179990	6.93G	45124190	6.77G	96.77	91.61	39813899
C_4h_2	46409128	6.96G	44910160	6.74G	97.27	92.62	40048934
C_4h_3	44321476	6.65G	43052624	6.46G	96.27	90.81	37441672
C_24h_1	44786892	6.72G	43110154	6.47G	95.85	90.05	37201612
C_24h_2	44943188	6.74G	43393564	6.51G	95.83	90.02	37413049
C_24h_3	45795096	6.87G	44886564	6.73G	97.42	92.92	39924459
SAL20_4h_1	46061926	6.91G	44890260	6.73G	97.06	92.29	39336186
SAL20_4h_2	45914726	6.89G	44580756	6.69G	97.22	92.65	39383060
SAL20_4h_3	50122024	7.52G	48685320	7.30G	95.69	89.74	42000224
SAL20_24h_1	42676610	6.40G	41583698	6.24G	97.26	92.68	36631715
SAL20_24h_2	47687056	7.15G	46363026	6.95G	97.36	92.84	40997032
SAL20_24h_3	46441770	6.97G	45115378	6.77G	97.41	92.91	39895803
